# Association of Medical Editors of the United States

**Published:** 1872-06

**Authors:** 


Association of Medical Editors of the United States. This Association held its third anniversary meeting on the morning of May 6th, in Horticultural Hall, Philadelphia—the President, Dr. B. F. Dawson, occupying the chair. A resolution was adopted offering $100 for the best essay on some subject, to be determined at each annual meeting, to be competed for by all medical editors. Drs. Dawson, Davis and Stone were appointed a committee to recommend a subject for the next essay, and to appoint a committee to decide upon the best which should be offered.

The Association reassembled, when they were addressed by the President, on the "Origin of Medical Science."

After the transaction of some minor business, the meeting adjourned.

